# Spanish norms focused on learning measures for the picture version of the free and cued selective reminding test with immediate recall

**DOI:** 10.1177/13872877251376906

**Published:** 2025-09-12

**Authors:** Andrea Luque-Tirado, Ernesto García-Roldán, Melisa González-Acosta, Andrea Herrera-Pozo, Julio Hernández Mendoza, Ángela Almodóvar-Sierra, Marta Marín-Cabañas, María Bernal Sánchez-Arjona, Juan Pedro Vargas-Romero, Emilio Franco-Macías

**Affiliations:** 1 Memory Unit, Department of Neurology, Virgen del Rocío University Hospital, Seville, Spain; 2 Biomedicine Institute of Seville IBiS, University Hospital Virgen del Rocio, CSIC, University of Seville, Seville, Spain; 3 Methodological and Statistical Support Unit, Fundación para la Gestión de la Investigación en Salud de Sevilla (FISEVI), Seville, Spain; 4 Department of Experimental Psychology, University of Seville, Seville, Spain

**Keywords:** Alzheimer's disease, free and cued selective reminding test, normative study, picture version, Spanish

## Abstract

**Background:**

The Free and Cued Selective Reminding Test (FCSRT) is a gold-standard among memory tests. Administration focused on learning measures may be more feasible for settings with limited face-to-face time per patient.

**Objective:**

To obtain norms based on learning measures for the picture version of the FCSRT with Immediate Recall (pFCSRT + IR) from Spanish population.

**Methods:**

A prospective normative study. Cognitively unimpaired volunteers were systematically recruited if eligible (age ≥50, no memory complaints, and a total TMA-93 score at or above the 10th percentile). In a second and independent session, the pFCSRT-IR was administered and its free recall and total recall (learning measures) were scored (range of score: 0–48 points). If a variable showed a high ceiling effect it was dichotomized; otherwise, a regression-based method was followed.

**Results:**

The final sample included 257 participants. Mean age and years of schooling were 67.46 (SD = 9.42, range = 50–88) years and 10.30 (SD = 5.81, range = 0–30) years, respectively. 68.9% were females. Scores for total recall showed ceiling effect, with 90.7% of the participants scoring > 46, which allowed dichotomizing the cutoff point at 46/47 (10th percentile). Free recall scores exhibited variability and were influenced by years of schooling, as well as by an age-by-sex interaction identified in the regression analysis.

**Conclusions:**

The study provides norms based on learning measures for using the pFCSRT + IR in Spain. The 46/47 cutoff point for total recall may be a reliable and easy-to-use measure for diagnosing memory impairment. Norms for free recall must also take an age-by-sex interaction into account.

## Introduction

The Free and Cued Selective Reminding Test (FCSRT) is considered a gold-standard among tests that evaluate episodic memory.^1^ The test controls attention and cognitive processing to optimize focus and mental engagement through semantic cues.^2^ A meta-analysis suggests that the diagnostic utility of tests based on cue efficiency is higher than that of tests using only free recall of word lists or story recall for assessing memory in patients with cognitive complaints.^3^

The FCSRT has been extensively validated for detecting early Alzheimer's disease (AD) and for discriminating AD from other types of dementia.^2,4,5^ Additionally, longitudinal studies have highlighted the role of the test in predicting future cases of dementia and identifying which patients with mild cognitive impairment (MCI) are at higher risk for developing AD,^6,7^ as well as those with prodromal AD who are likely to rapidly convert to dementia.^8^

The FCSRT has different versions: one uses pictures to represent test items (“picture version”),^9^ while the other uses printed words (“word version”).^10^ The picture version (pFCSRT) is the original one.^2^ The two versions are not equivalent,^11^ as the pFCSRT has the advantage of dual encoding^12^: pictures evoke both a verbal and a visual representation, whereas words only elicit a verbal representation in the memory process. Therefore, norms for each version are not interchangeable.^11^

On the other hand, the administration of the FCSRT, following the guidelines established by its authors,^13^ consists of three phases: an initial learning phase with cue encoding; three trials, each preceded by a distraction task involving counting backward for 20 s, during which free recall is tested or, if unsuccessful, cued recall is attempted, or if still unsuccessful, the item and its cue are presented together again (“selective reminding”).^13^ Finally, there is a delayed recall phase, conducted 30 min later, involving both free recall and cued recall.^13^ Based on this, there have been variations, such as the introduction of an immediate recall with cues during the controlled learning phase. This variation is known as the FCSRT with Immediate Recall (FCSRT + IR).

The FCSRT produces several results of interest. From the learning phase, a free recall score is obtained, which is the sum of the scores from the three trials, along with a total recall score, which is the sum of the free recall and cued recall scores across the three trials. From the retention phase, both free recall and total recall (the sum of free recall and cued recall) can be obtained in the final delayed trial, conducted 30 min after the third trial of the learning phase.^13^ Learning measures have been predominantly used in two research contexts: observational cohort studies for the prediction of MCI and dementia^14^; and clinical trials involving anti-amyloid therapy,^15,16^ either during candidate screening,^15^ or as an efficacy measure within cognitive composite outcomes.^16^ Interestingly, learning measures have been demonstrated to be as predictive as retention measures for the risk of incident MCI in patients initially without cognitive impairment, as part of longitudinal observational cohorts.^13^ The focus on these learning measures may have a feasibility advantage in contexts with limited time available for assessing patients’ memory.

In Spain, normative data are currently available only for the verbal version of the FCSRT,^17,18^ with existing studies primarily focusing on retention measures^17^ or total recall scores.^18^ Developing normative data centered on learning measures—including both free and total recall—would be of particular interest, especially if it incorporated a pictorial variant. This would enable comparisons with the American population, where the pictorial version is predominantly used.^13,14^ Such a study would provide a foundation for future longitudinal research and support the inclusion of patients in AD clinical trials that employ the picture version of the FCSRT. Furthermore, normative data for this pictorial version, more accessible to individuals with limited reading literacy, would enhance the utility of the test for both clinical and research purposes in populations with greater educational diversity, such as that of Spain.

Thus, the aim was to obtain norms, focused on learning measures, for the picture version of the FCSRT + IR (pFCSRT + IR) in the Spanish population.

## Methods

### Permission

Permission was obtained from the Albert Einstein College (AEC) to conduct this normative study. The original material for administrating the pFCSRT + IR was provided by the AEC. This material had been specifically designed for the Spanish population by the AEC and consists of three equivalent forms: A, B, and C. We chose to work with Form A.

### Design

We conducted a cross-sectional, observational, and prospective normative study of the pFCSRT + IR among older Spanish adults.

### Study population

Participants were recruited from cognitively unimpaired volunteers attending the general neurology outpatient clinic at the University Hospital Virgen del Rocio in Seville, Spain. They were individuals who either sought consultation for non-cognitive complaints or were cognitively unimpaired companions of patients visiting the center. The vast majority of participants were companions of patients. Only patients who sought consultation for conditions such as syncope, primary headaches, disc herniation, carpal tunnel syndrome, meralgia paresthetica, and peripheral facial paralysis—which are clearly unrelated to cognitive impairment and showed no evidence of structural lesions on brain neuroimaging—were invited to participate.

Inclusion criteria were: 1) age 50 years or older; 2) score of 1 on Global Deterioration Scale, no memory complaints; absence of obvious memory disorder in the clinical interview; 3) no memory impairment, defined as a score at or above the 10th percentile on the Memory Associative Test of the district of Seine-Saint-Denis (TMA-93)^19,20^; 4) capable of independent living.

The following were the exclusion criteria: 1) history of neurological disease potentially causing cognitive impairment: 2) significant systemic disease that could affect cognitive evaluation; 3) poor vision or hearing despite correction; 4) severe psychiatric disorders (e.g., active major depression, schizophrenia, or bipolar disorder); 5) use of psychotropic medications, and/or a history of psychoactive substance use.

Age, gender, and years of schooling were considered as socio-demographic variables. Recruitment was systematic, with eligible individuals consecutively invited to participate.

Recruitment was closed once two conditions were met: the sample provided broad representation across the full spectrum of age and years of schooling, and the total number of participants substantially exceeded the minimum of 100 required for a regression-based normative study.^21,22^

### Procedures: TMA-93 and pFCSRT + IR

*TMA-93.* The TMA-93 was administered to assess memory status and determine eligibility. Administration and scoring (maximum score: 30) followed the instructions provided by its authors.^23^ Participants who scored at or above the 10th percentile on the TMA-93, according to the Spanish normative data,^19,20^ were considered to have preserved memory and eligible for this normative study.

*pFCSRT* *+* *IR.* Eligible participants were scheduled for an independent session to administer the pFCSRT + IR. This session took place between two and six weeks after eligibility was confirmed by the TMA-93.

The form A of the pFCSRT + IR was administered following instructions provided by the AEC.^13^ Briefly, the test begins with an encoding phase, in which participants examine 16 easily recognizable drawings, each representing a unique semantic category. The items are presented in groups of four, on four different cards. The patient is asked to point and name each item after its corresponding semantic cue. After the right identification, the card is removed, and the immediate cued recall of the four items is tested. The participant is reminded of any items they failed to recall by presenting the cue along with the item. Once the immediate recall for each card is completed, the participant undergoes three recall trials, each preceded by 20 s of backward counting as interference. Each recall trial consists of three parts. First, each participant has up to 2 min to freely recall as many items as possible. Next, semantic cues are verbally provided for the items not retrieved. Finally, if the participant fails to recall an item with the category cue, s/he is reminded by presenting the cue along with the item (selective reminding).^13^ Based on feasibility criteria, we focused on learning measures by excluding the 30-min delayed free and cued recall from the FCSRT. In this sense, we recorded two results: the free recall, the sum of free recall from the three memory trials, and the total recall, the sum of both free and facilitated recall across the three memory trials. Scores for both variables can range from 0 to 48.^13^

### Statistical study

We present descriptive results as absolute and relative frequencies for categorical variables and measures of central tendency and variability for quantitative variables.

**Figure 1.**
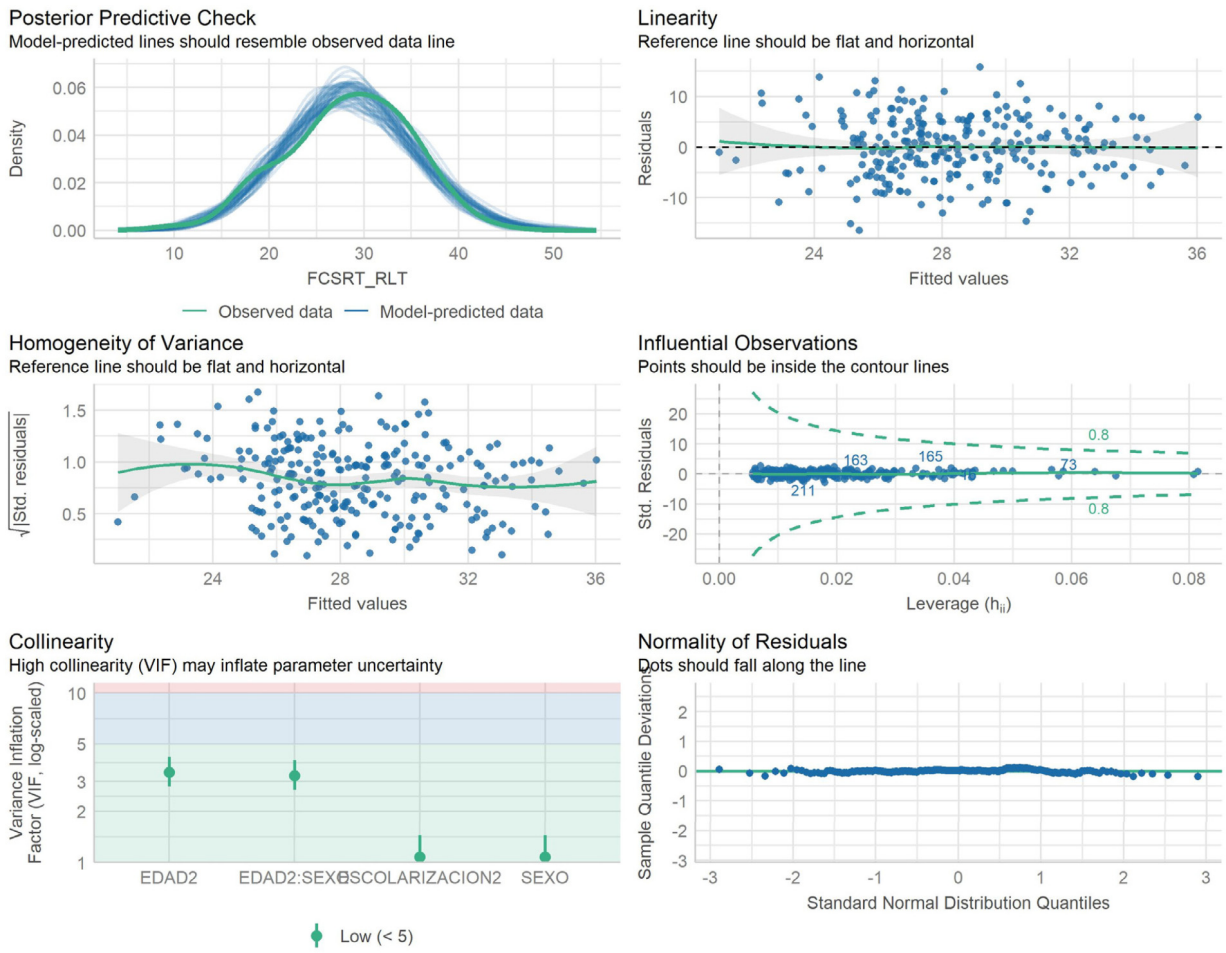
The assumptions of the model—linearity, normality of residuals, independence of residuals, absence of multicollinearity, and absence of influential values—were verified, except homoscedasticity.

To establish the normative data, we use a dual approach. Total recall showed a high ceiling effect, so it was dichotomized, while a regression-based method was applied to the free recall. In this last case, we centered age and years of schooling (we considered years of schooling as a continuous variable in this approach) and constructed a multiple linear regression model with free recall as the dependent variable, following the methodology proposed by Van der Elst (2024).^22^ Initially, a full linear regression model was constructed including age (centered), years of schooling (centered) and sex as main effects, all two-way interactions among these variables, and the quadratic and cubic terms for the continuous variables (age and years of schooling). A backward stepwise selection procedure based on the p-value of the beta coefficients was applied to remove non-significant predictors, using a conservative significance threshold of α = 0.01 due to the number of contrasts. When the homoscedasticity assumption was violated, robust standard errors were used. Variables were eliminated in the following order: two-way interactions, cubic and quadratic terms, and finally main effects. If any interaction or higher-order term remained significant, the corresponding lower-order terms were retained in the model regardless of their individual p-values. The final model included centered age, centered education, sex, and the age-by-sex interaction. The assumptions of the model—linearity, normality of residuals, homoscedasticity of residuals, independence of residuals, absence of multicollinearity, and absence of influential values—were verified (Figure 1), but homoscedasticity was unmet (Breusch-Pagan test p-value = 0.019). For creating the percentiles tables, we followed step-by-step procedures outlined in the reference source.^22^ The constant and coefficients obtained in the model were used to compute predicted scores from the regression equation. Residuals were calculated by subtracting each predicted score from the observed scores. Subsequently, the residuals were converted into z-scores. Since homoscedasticity was not verified, the residual variance was modeled as a function of the fitted values using first-, second-, and third-degree polynomial regression models. The first-degree polynomial regression was selected based on the lowest Akaike Information Criterion (AIC) and comparable performance to the higher-degree models. To standardize residuals, each was divided by the estimated standard deviation obtained from the selected variance prediction function. Finally, the z-scores were converted into percentile values using the standard normal cumulative distribution function, since the model assumption of normality of the residuals was verified. This procedure provided the percentile associated with an observed score.

Statistical significance was set at p < 0.05, and 95% confidence intervals were calculated.

The analysis was performed using R software version 4.4.1 (R Core Team, 2024). The following R packages were used: haven (version 2.5.4), lmtest (version 0.9–40), sandwich (version 3.1–0), performance (version 0.12.4) and NormData (version 1.1). Multiple linear regression was fitted using the lm() function. To assess the assumptions of the model, we used the check_model() function from the performance package, along with its associated functions: check_normality(), check_heteroscedasticity(), check_autocorrelation(), check_collinearity(), and check_outliers(). To work with robust standard errors, the functions coeftest() from the lmtest package and vcovHC() from the sandwich package were used. Once the final model was constructed, the Stage.1() function from the NormData package was used to construct and compare the variance prediction functions, applying the corresponding summary() and plot() functions for select the optimal variance model.

## Results

**Figure 2.**
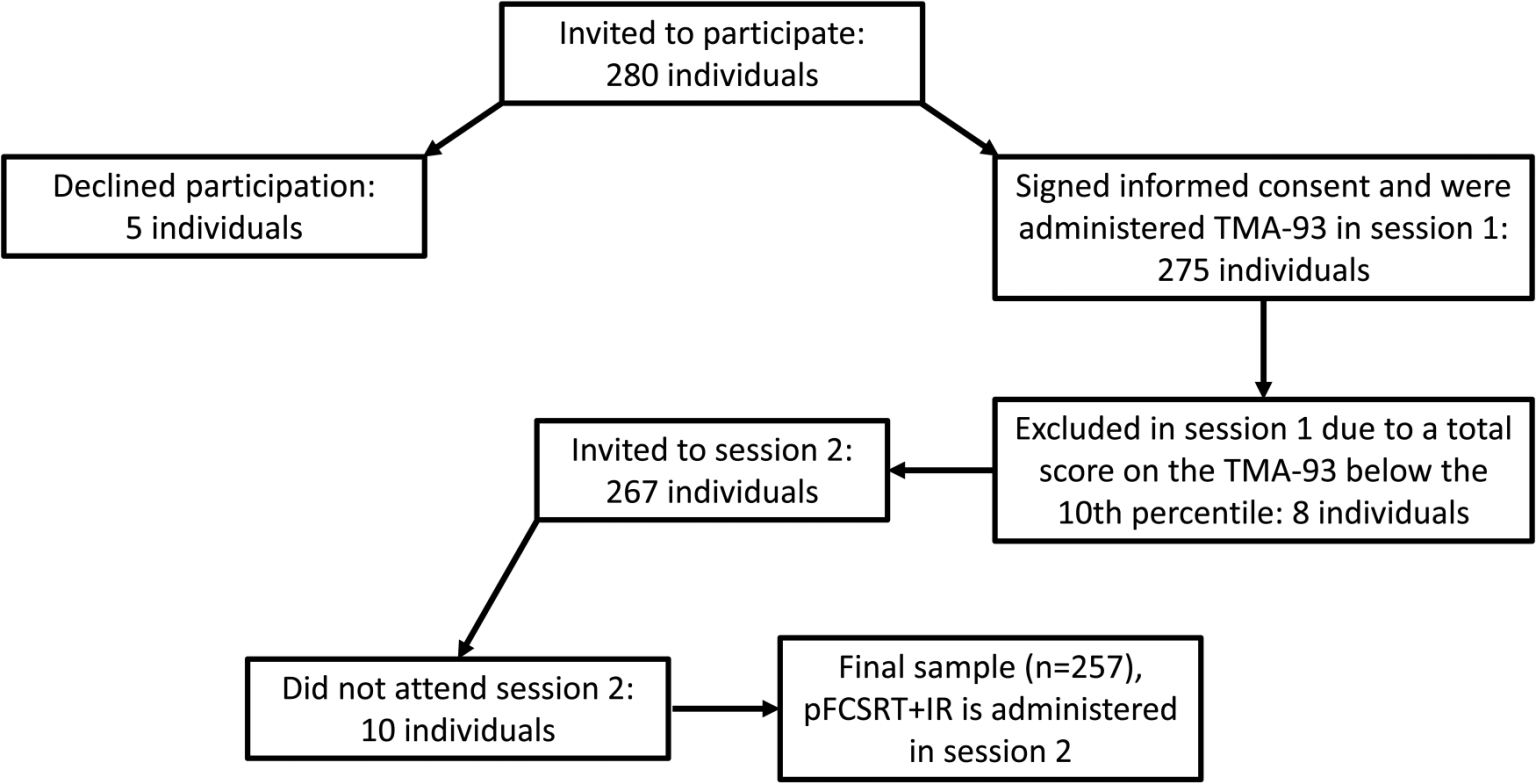
Study flow chart.

Between October 2023 and April 2024, a total of 280 individuals were invited to participate in the study. Among them, five declined to participate. The TMA-93 was administered to the remaining 275 participants; 8 of these scored below the 10th percentile and were excluded in accordance with the study protocol. Additionally, 10 participants did not attend the independent second session, during which the pFCSRT + IR was administered. Consequently, the final sample consisted of 257 individuals. The study flow chart is presented in Figure 2.

Table 1 presents the distribution of socio-demographic variables in the final sample. Regarding age, the sample had a median of 68 years (interquartile range: 60–75; range: 50–88; mean: 67.46; SD: 9.42), with adequate representation of older age groups: 39.7% were over 70 years old, and 19.5% were over 76 years-old (Table 1). Females (68.9%) outnumbered males, reflecting their traditional caregiving role of partners in Spanish society (Table 1). In terms of education, the median years of schooling was 9 (interquartile range: 6–13; range, 0–30; mean: 10.30; SD: 5.81) (Table 1). 44 participants (17.1%) did not complete primary education. Of these, 9 never attended school. The older segments, particularly females, were less educated, a common characteristic of the Spanish population.

**Table 1.** Socio-demographic characteristics of the sample.

Total Sample (n = 257, 100%)		Males (n = 80, 31.1%)		Females (n = 177, 68.9%)	
Age (y)	Years of Schooling	Age (y)	Years of Schooling	Age (y)	Years of Schooling
<60 (n = 60, 23.3%)	≤7 (n = 16, 6.2%)	<60 (n = 11,4.3%)	≤7 (n = 3, 1.2%)	<60 (n = 49, 19%)	≤7 (n = 13.1%)
	8–12 (n = 21, 8.2%)		8–12(n = 2, 0.8%)		8–12 (n = 19, 7.4%)
	≥13 (n = 23, 8.9%)		≥13, (n = 6, 2.3%)		≥13 (n = 17, 6.6%)
60–65 (n = 50, 19.5%)	≤7 (n = 12, 4.7%)	60–65 (n = 16, 6.2%)	≤7 (n = 1, 0.4%)	60–65 (n = 34, 13.2%)	≤7 (n = 11, 4.3%)
	8–12 (n = 19, 7.4%)		8–12(n = 6, 2.3%)		8–12 (n = 13, 5.1%)
	≥13 (n = 19, 7.4%)		≥13, (n = 9, 3.5%)		≥13 (n = 10, 3.9%)
66–70 (n = 45, 17.5%)	≤7 (n = 16, 6.2%)	66–70 (n = 14, 5.4%)	≤7 (n = 3, 1.2%)	66–70 (n = 31, 12%)	≤7 (n = 13, 5%)
	8–12 (n = 12, 4.7%)		8–12(n = 5, 1.9%)		8–12 (n = 7, 2.7%)
	≥13 (n = 17, 6.6%)		≥13, (n = 6, 2.3%)		≥13 (n = 11, 4.3%)
71–76 (n = 52, 20.2%)	≤7 (n = 28, 10.9%)	71–76 (n = 15, 5.8%)	≤7 (n = 5, 1.9%)	71–76 (n = 37, 4.3%)	≤7 (n = 23, 8.9%)
	8–12 (n = 15, 5.8%)		8–12(n = 6, 2.3%)		8–12 (n = 9, 3.5%)
	≥13 (n = 9, 3.5%)		≥13, (n = 4, 1.5%)		≥13 (n = 5, 1.9%)
>76 (n = 50, 19.5%)	≤7 (n = 16, 6.2%)	>76 (n = 24, 9.3%)	≤7 (n = 7, 2.7%)	>76 (n = 26, 10.1%)	≤7 (n = 9, 3.5%)
	8–12 (n = 26 10.1%)		8–12 (n = 12, 4.7%)		8–12 (n = 14, 5.4%)
	≥13 (n = 8, 3.1%)		≥13 (n = 5, 1.9%)		≥13 (n = 3, 1.2%)

Percentages represent the proportion of each group relative to the total sample.

The TMA-93 total score exhibited a ceiling effect, with a median of 30 (interquartile range: 29–30; range: 20–30), consistent with a sample of cognitively unimpaired participants.

For the pFCSRT + IR, the total recall also displayed a ceiling effect, with a median score of 48 (interquartile range: 47–48; range, 33–48). As shown in Table 2, only 9.3% of individuals scored below 47, while 73.5% achieved the maximum score of 48, and 17.1% scored 47 points (Table 2). This result enables us to dichotomize the total recall using a cut-off 46/47, corresponding to the 10th percentile.

**Table 2.** Distribution of scores for the pFCSRT + IR total recall.

Total Score	Frequency	Percentage	Cumulative Percentage
33	1	0.4%	0.4%
41	1	0.4%	0.8%
42	2	0.8%	1.6%
44	1	0.4%	1.9%
45	8	3.1%	5.1%
46	11	4.3%	9.3%
47	44	17.1%	26.5%
48	189	73.5%	100%

In contrast, the pFCSRT + IR free recall showed greater variability. Specifically, 14% of individuals scored below 21, 30.4% scored below 25, and only 39.7% achieved scores above 30 points (Table 3). This result prompted the use of multiple regression analysis. As explained in the Statistical Study section, centered age, centered years of schooling, sex, and the interaction between centered age and sex were included in the final regression model (Table 4). The coefficient associated with years of schooling was 0.17 (p = 0.007) indicating that a 1-point difference in expected free recall corresponds to approximately 6 years of schooling, assuming all other variables remain constant. However, the effect of age varies by sex, as the interaction between age and sex was statistically significant (coefficient = -0.25, p = 0.002). Although the individual coefficients for age and sex were not statistically significant (coefficients = -0.05 and 1.23; p = 0.406 and 0.124, respectively), they had to be retained in the model due to the presence of this interaction. Interpreting the coefficients based on their statistical significance, age did not affect men, whereas it did affect women. However, all coefficients were used in the calculation of predicted values, and therefore an interpretation was provided for each of them. The coefficient associated with sex was 1.23, meaning that, taking the mean age as a reference, women scored 1.23 points higher than men. When the interaction term was taken into account, for each year above the mean age, women's scores decreased by 0.30 points (−0.25 from the interaction plus −0.05 from the main effect of age), whereas for men, the decrease is only 0.05 points. In other words, for women, a 1-point difference in expected free recall corresponded to approximately 4 years of age, while for men, it corresponds to around 20 years, assuming years of schooling remain constant.

**Table 3.** Distribution of scores for the pFCSRT + IR free recall.

Total Score	Frequency	Percentage	Cumulative Percentage
9–15	6	2.3%	2.3%
16–20	30	11.7%	14%
21–25	42	16.3%	30.4%
26–30	77	30%	60.3%
31–35	65	25.3%	85.6%
36–40	33	12.8%	98.4%
>40	4	1.6%	100%

**Table 4.** Final regression model for the free recall score.

	β±Std. Error	*t*	*p*
Constant	27.38 ± 0.65	41.8853	<0.001
Age (centered)	−0.05 ± 0.06	−0.8331	0.406
Years of schooling (centered)	0.17 ± 0.06	2.71	0.007
Sex (0 male, 1 female)	1.23 ± 0.80	1.54	0.124
Age (centered) * Sex	−0.25 ± 0.08	−3.16	0.002

Adjusted R^2^ = 0.1699.

The constant and coefficients obtained in the model were then used to compute predicted scores from the regression equation:

Predicted Score = 27.38- 0.05 * (Age - 67.46) + 0.17 * (years of schooling – 10.30) + 1.23 * Sex – 0.25 * (Age - 67.46) * Sex

For creating the percentiles tables we followed step-by-step the procedures described on the methods. Since the assumption of homoscedasticity was not met, the residual variance was modeled as a function of the fitted values using a first-degree polynomial regression model (Table 5). This model must be taken into account when calculating the z-score.

**Table 5.** Residual variance model.

	β
Intercept	106.86
Fitted values	−2.55

The Supplemental Material includes a calculator to estimate the percentile corresponding to a raw score, taking into account age, years of schooling, and sex, and provides the normative tables with raw scores equivalence to percentiles for free recall. Age and years of schooling are entered in their original scales because they are automatically centered during the calculation process. The sex variable must be coded as 0 = male and 1 = female.

## Discussion

This normative study for the pFCSRT + IR, focused on learning measures and conducted with cognitively unimpaired individuals, provides reference values tailored for Spanish population. These norms will be instrumental in assessing patients with memory complaints using the test.

The study addresses a gap previously identified in Spain, since norms were available for the word version of the test,^17,18^ but not for the picture one, which are not equivalent.^11^ This picture version is particularly suitable for older individuals with low education levels or reading difficulties, who make up a significant proportion of the elderly population in Spain. We provide norms for a broad spectrum of educational levels, including the least educated.

This study intentionally focuses on the learning measures of the FCSRT. These measures are obtained during the test's learning phase.^13^ Learning measures are scored based on the sum of free recall across the three trials (free recall score) and the total recall score (the combined total of free recall and cued recall from the same three trials).^13^ The focus on learning measures is justified by their demonstrated predictive value, which is comparable to that of retention measures (such as delayed free and total recall) in identifying MCI.^14^ Importantly, learning measures do not require the 30-min delay necessary for delayed recall testing,^13^ enhancing feasibility in time-constrained clinical settings. Learning measures have also been the basis for the predictive Stages of Objective Memory Impairment paradigm in the FCSRT,^7^ and, additionally, they have had practical utility in AD clinical trials.^15,16^

From this point onward in the discussion, when we refer to free recall and total recall, we are referring to those obtained during the learning phase.

In this Spanish normative study, total recall showed a ceiling effect. Only 9.3% of individuals scored below 47, while 73.5% achieved the maximum score of 48, and 17.1% scored 47 points. This ceiling effect for total recall has been consistently observed in cognitively intact individuals in normative or validation studies across various populations.^24–26^

This result enabled us to dichotomize the total recall using a cut-off at 46/47, corresponding to the 10th percentile. This cutoff is further reinforced by findings from previous studies.^7,27–29^ On the one hand, in longitudinal studies of the American population, a score below 47 marked the threshold for MCI.^7^ In fact, this cutoff has already been adopted as an indicator of memory impairment in some clinical trials.^27^ On the other hand, in a preclinical AD study, this 46/47 cutoff in total recall was associated with greater risk of progression to global Clinical Dementia Rating of 0.5 for 2 sequential years.^29^ Finally, from a local perspective, this cutoff replicates the best one obtained in the Spanish preliminary validation of the pFCSRT + IR for distinguishing 55 patients with amnestic MCI from 69 healthy controls.^28^ Although convenience sampling was used, resulting in a high prevalence of amnestic MCI (44%), which may limit the generalizability of the findings,^28^ in that study, the diagnostic utility of total recall was good (AUC = 0.86; 95% CI = 0.79–0.93), with a cutoff score of 46/47 achieving a sensitivity of 80% and a specificity of 88.4%.^28^ We propose that this cutoff score be used to diagnose memory impairment in patients who consult for memory complaints. Although cultural differences require validation and normative studies for each population to define the optimal cutoff points, the similarity with the results obtained in the American population leads us to suspect that this 46/47 cutoff may be quite robust cross-culturally. Another advantage of using the 46/47 cutoff for the total recall for diagnosing memory impairment by the pFCSRT + IR is its ease of assessment, eliminating the need to consult normative tables.

The free recall scores exhibited significant variability, influenced by years of schooling, and an age-by-sex interaction. What the model actually shows is that age affects women and men differently. Therefore, even though age alone may not appear significant, it is shown to be influenced by sex. Taking this into account, the influence of age on the score is greater in women (−0.30) than in men (−0.05). In fact, this would suggest that age has no effect in men, but it does in women. Thus, in the case of women, the effect of one year of age is greater than the effect of one year of schooling. In men, it would be the opposite, especially considering that the effect of age is not statistically significant in this group. The normative tables confirm that women outperform men in the younger age groups; however, as age increases, their scores decline more sharply than those of men, whose performance remains more stable and ultimately surpasses that of women in older age groups. Several conclusions and hypotheses can be derived from this interesting result. On one side, when normative studies restrict regression analyses to only the main sociodemographic variables and fail to account for interactions between them, the results may lead to misleading conclusions and obscure meaningful patterns in the normative data. To date, normative studies of the pFCSRT have not found a significant effect of gender on test scores. However, these studies have not examined potential interactions among key sociodemographic variables.^24,30,31^ On the other side, the finding that women outperform men- while men's performance remains more stable across age ranges- has been reported in previous normative studies of other episodic memory test, as the Rey Auditory Verbal Learning Test,^32^ and has been associated with gender-related differences in encoding and consolidation strategies.^33,34^

Longitudinal studies conducted on the American population with serial administration of the pFCSRT + IR have shown that, in cognitively unimpaired older individuals who eventually develop dementia, regarding learning measures, the free recall declines up to 5 years before diagnosis, indicating incident cognitive impairment.^7^ In contrast, the total recall declines only 2 years before diagnosis, reflecting prevalent cognitive impairment.^7^ An interesting finding is that if we apply the American longitudinal data,^7^ which indicate that a free recall score below 30 signals a risk of incident cognitive impairment for individuals with a total recall score above 46, nearly 60% of this cognitively unimpaired Spanish sample would be at risk. However, this conclusion is not valid as those results, which may have a cultural bias, should not be extrapolated from one population to another. Looking at specific comparisons,^7^ the cohort from the Einstein Aging Study had more years of education (mean = 13.5; standard deviation = 3.5) than the participants in our study (mean = 10.3; standard deviation = 5.8). Consequently, longitudinal studies in the cognitively unimpaired older Spanish population are needed to account for variability in free recall scores and accurately define cutoff points that estimate the risk of incident cognitive impairment using the pFCSRT + IR. Additionally, it is now clear that such studies must account for both years of schooling and the age-by-sex interaction.

A key strength of this study lies in its prospective and consecutive recruitment of participants over seven months under typical clinical practice conditions, which enhances its pragmatic approach.

A limitation of this study is that the sample may not be fully representative of the Spanish population, as recruitment was limited to cognitively unimpaired volunteers aged 50 and older who attended a general neurology outpatient clinic in a specific city in southern Spain. However, this recruitment strategy ensured the inclusion of individuals with diverse educational backgrounds, all within the age group at risk for AD. Another limitation could be the relatively small size sample. However, the regression-based norming method used for the pFCSRT + IR free recall employs a regression model with covariates to estimate a normal distribution. This approach enables smaller samples to generate norms more effectively than traditional methods.^20,21,35^ Additionally, the final sample in this study exceeds that of other recent normative studies based on regression for the FCSRT.^18,26,36^ Another potential limitation was the absence of a comprehensive neuropsychological battery to rule out cognitive impairment in the patients. Instead, we used the TMA-93 to assess participants’ cognitive status and determine their eligibility. The TMA-93, validated by biomarkers for early AD diagnosis,^37^ has extensive normative data for the Spanish population.^19,20^ This test evaluates visual relational binding, assessing memory and language,^23^ two cognitive domains closely linked to early AD and that should be preserved in individuals considered cognitively unimpaired. As anticipated, total scores on the TMA-93 demonstrated a ceiling effect. Lastly, separating the procedures into two independent sessions—one for the TMA-93 and another for the pFCSRT + IR- resulted in an additional loss of 10 participants who did not attend the second session. However, this separation was necessary to prevent interference between the two tests which share pictorial and semantic approaches.

In conclusion, this study provides norms for assessing the learning measures of the pFCSRT-IR in Spain, addressing a need that memory evaluation had in our country. This abbreviated and picture version of the FCSRT would be more feasible for illiterate older people and in settings with limited time for examination, two prevalent conditions that limit memory evaluation in Spain. The 46/47 cutoff for the total recall seems to be robust and easy to apply for diagnosing memory impairment. It should be validated by specific biomarkers for diagnosing prodromal AD in Spanish population. For predicting the risk of developing cognitive impairment based on free recall, longitudinal studies should be conducted in older population, considering variables as years of schooling and the age-by-sex interaction.
